# A novel conserved mechanism for plant NLR protein pairs: the “integrated decoy” hypothesis

**DOI:** 10.3389/fpls.2014.00606

**Published:** 2014-11-25

**Authors:** Stella Cesari, Maud Bernoux, Philippe Moncuquet, Thomas Kroj, Peter N. Dodds

**Affiliations:** ^1^Institut National de la Recherche Agronomique, Unité Mixtes de Recherche Biology and Genetics of Plant-Pathogen InteractionsMontpellier, France; ^2^Centre de Coopération Internationale en Recherche Agronomique pour le Développement, Unité Mixtes de Recherche Biology and Genetics of Plant-Pathogen InteractionsMontpellier, France; ^3^Agriculture Flagship, Commonwealth Scientific and Industrial Research OrganisationCanberra, ACT, Australia; ^4^Commonwealth Scientific and Industrial Research Organisation, Digital Productivity and ServiceCanberra, ACT, Australia

**Keywords:** NLR protein pairs, integrated decoy, pathogen recognition, plant immunity, *Arabidopsis thaliana*, rice

## Abstract

Plant immunity is often triggered by the specific recognition of pathogen effectors by intracellular nucleotide-binding, leucine-rich repeat receptors (NLR). Plant NLRs contain an N-terminal signaling domain that is mostly represented by either a Toll-interleukin1 receptor (TIR) domain or a coiled coil (CC) domain. In many cases, single NLR proteins are sufficient for both effector recognition and signaling activation. However, many paired NLRs have now been identified where both proteins are required to confer resistance to pathogens. Recent detailed studies on the *Arabidopsis thaliana* TIR-NLR pair RRS1 and RPS4 and on the rice CC-NLR pair RGA4 and RGA5 have revealed for the first time how such protein pairs function together. In both cases, the paired partners interact physically to form a hetero-complex receptor in which each partner plays distinct roles in effector recognition or signaling activation, highlighting a conserved mode of action of NLR pairs across both monocotyledonous and dicotyledonous plants. We also describe an “integrated decoy” model for the function of these receptor complexes. In this model, a plant protein targeted by an effector has been duplicated and fused to one member of the NLR pair, where it acts as a bait to trigger defense signaling by the second NLR upon effector binding. This mechanism may be common to many other plant NLR pairs.

## Introduction

Plants, unlike animals do not possess circulating immune cells to intercept microbial signals. Thus, their innate immune system is cell autonomous, with each plant cell able to recognize microbial signals and respond accordingly. To prevent infection by viruses, bacteria, oomycetes, fungi or nematodes, plant possess two main types of immune receptors: pattern recognition receptors (PRRs) on the cell surface and intracellular nucleotide-binding and leucine-rich repeat receptors (NLR) (Monaghan and Zipfel, 2012; Jacob et al., 2013; Qi and Innes, 2013). PRRs are involved in the recognition of so called pathogen-associated molecular patterns (PAMPs) which are conserved molecules characteristic of entire groups of microbes, whereas NLR resistance (R) proteins perceive specific effectors called avirulence proteins (AVR) derived from pathogens (Jones and Dangl, 2006; Dodds and Rathjen, 2010). Such perception activates effector-triggered immunity that is often associated with a localized programmed cell death called the hypersensitive response (HR). NLR proteins are multidomain proteins and possess a conserved architecture including a C-terminal leucine-rich repeat (LRR) domain, a central nucleotide-binding (NB) domain and a variable N-terminal domain (Takken and Goverse, 2012). In most cases NLR proteins possess a Toll/interleukin-1 receptor (TIR) or coiled-coil (CC) domain at their N-terminus defining two major classes of R proteins: TIR-NLRs and CC-NLRs (Meyers et al., 1999; Pan et al., 2000).

Flor's classical gene-for-gene concept of plant disease resistance states that “for each gene that conditions resistance in the host (*R* gene) there is a corresponding gene that conditions pathogenicity in the parasite (*AVR* gene)” (Flor, 1971). Indeed many cases of matching *R* and *AVR* genes have been described and, in most cases, individual NLR proteins mediate AVR effector recognition and confer resistance (Jones and Dangl, 2006; Bernoux et al., 2011a). However, an emerging picture is that disease resistance against a pathogen isolate, or recognition of an AVR protein requires, in certain cases, complementary pairs of NLR genes (Eitas and Dangl, 2010). In the first demonstration of this, two TIR-NLR proteins, RPP2A and RPP2B, were both shown to be necessary for *Arabidopsis thaliana* resistance to the oomycete pathogen *Hyaloperonospora arabidopsidis* (Sinapidou et al., 2004). More recently, a second pair of Arabidopsis TIR-NLR proteins, RPS4 and RRS1, was shown to be required for recognition of AvrRps4 from the bacterial pathogen *Pseudomonas syringae*, PopP2 from *Ralstonia solanacearum* and an as yet uncharacterized factor produced by the fungal pathogen *Colletotrichum higginsianum* (Gassmann et al., 1999; Deslandes et al., 2003; Birker et al., 2009; Narusaka et al., 2009). This example demonstrates that a single complementary pair of R proteins can mediate recognition of multiple pathogens. Complementary pairs of distinct CC-NLR proteins can also confer resistance as shown by the wheat Lr10 and RGA2 protein pair that mediate resistance to wheat leaf rust caused by *Puccinia triticina* (Loutre et al., 2009), barley Rpg5 and RGA1 that confer resistance to *Puccinia graminis* (Wang et al., 2013) and melon Prv and Fom-1 that function against *Fusarium oxysporum* and *Papaya ring-spot virus* (Brotman et al., 2012). Recent studies have also described several rice CC-NLRs acting as functional pairs to mediate resistance against *M. oryzae*, including RGA4/RGA5, Pik-1/Pik-2, and Pi5-1/Pi5-2 (Ashikawa et al., 2008; Lee et al., 2009; Okuyama et al., 2011; Yuan et al., 2011; Zhai et al., 2014). Thus, NLR pairs have been shown to function in the recognition of bacterial, viral, oomycete and fungal pathogens in both monocotyledonous and dicotyledonous plants, suggesting this is a common and widespread mechanism in plant immunity.

Interestingly, all of these resistance loci comprise two tightly linked NLR coding genes, most of which (except *RPP2A/RPP2B*) are transcribed in opposite directions with a relatively short intergenic region. This conserved genomic organization could be important for co-regulation of these *NLR* genes or to prevent recombination events leading to separation or inappropriate pairing of NLRs which could cause loss of resistance or spontaneous necrosis (Bomblies et al., 2007). Receptor-like kinase genes of the *Lrk* (Lr10 receptor-like kinase) and *Tak* (*Triticum aestivum* kinase) family also occur in pairs in barley (Hu and Wise, 2008) and wheat (Feuillet et al., 2001), possibly for similar reasons. A key question raised is how do two distinct NLR proteins act together to mediate AVR protein recognition? In the present review, we describe new insights into the functional mechanisms of paired NLR proteins focusing on the recently published models of RPS4/RRS1 and RGA4/RGA5. We will also introduce and discuss a novel model for the recognition of AVR proteins by pairs of co-acting NLRs: the “integrated-decoy model.”

## Plant NLRs play distinct roles within co-acting pairs to confer resistance

In the absence of pathogens, NLR proteins are kept in an inactive state, whereas, after AVR-recognition, they are activated and induce disease resistance signaling. The molecular mechanisms occurring during the transition from “inactive” to “active” state and the downstream signaling partners are still largely unknown. However, since the first molecular characterization of an NLR-coding gene 20 years ago (Bent et al., 1994; Mindrinos et al., 1994), structure-function analyses have shown that individual domains and particular motifs of NLR proteins play critical roles in their auto-inhibition or activation mechanisms (Takken and Goverse, 2012; Qi and Innes, 2013). Current models of NLR function predict that, in the resting state, intramolecular interactions between different domains maintain R proteins in a closed auto-inhibited conformation. For a number of R proteins, this “off” state has been shown to be associated with ADP binding. It has been proposed that pathogen effector recognition favors a more open NLR protein structure allowing nucleotide exchange and binding to ATP (Williams et al., 2011). Since isolated TIR and CC domains have been shown to be sufficient to trigger an HR-like response (Maekawa et al., 2011; Bernoux et al., 2011b), it is proposed that in the activated state of NLRs, these N-terminal domains would become exposed to interact with signaling partners and initiate disease resistance responses. Structure-function analysis of the L6 TIR domain of flax and the MLA CC domain of barley showed that homotypic interactions of these signaling domains play an important role in the induction of HR (Bernoux et al., 2011b; Maekawa et al., 2011). However, these models based on NLRs functioning singly do not explain how two distinct NLR proteins co-operate in pathogen recognition and signaling.

Two recent studies provided the first analysis of how paired NLRs function together (Cesari et al., 2014; Williams et al., 2014). In one case, Williams et al. (2014) demonstrated by coupling crystal structure and functional analyses, that RPS4 and RRS1 TIR domains form homo- and hetero-dimers through a common conserved interface which includes a core serine-histidine (SH) motif. Mutation of the SH motif in the TIR domain of RRS1, RPS4 or both proteins abolished TIR hetero-dimer formation in solution and also prevented TIR homo-complex formation in yeast two-hybrid experiments. However, similar SH mutations introduced in the full length RPS4 and RRS1 did not prevent hetero-complex formation in co-immunoprecipitation experiments showing that other domains of the proteins, apart from the TIR domains, might also contribute to heterotypic interactions. Transient expression assays in tobacco revealed that the RPS4 TIR domain triggers an effector-independent cell death, which is dependent on the SH motif. Furthermore, a mutation within the RPS4 TIR domain (R30A) that strengthened its homodimerization also enhanced its autoactivity, indicating that the RPS4 TIR domain signals as a homo-dimer. Co-expression of the RRS1 TIR domain inhibited this RPS4 TIR autoactive cell death, and this was dependent on the RRS1 SH motif. This suggests that an inactive RRS1/RPS4 TIR hetero-dimer competes with formation of the active RPS4 TIR homo-dimer to inhibit signaling. Indeed, the structural data showed that both complexes involve a common interface and *in vitro* and *in vivo* experiments indicated that TIR domain hetero-dimers are more stable than homodimers. Thus, TIR domain hetero-interactions may play a crucial role in maintaining the full-length protein pair complex in an inactive state. Interestingly, TIR domain hetero-complex formation also seems to be required for AVR recognition since mutation of the SH motif in full length RRS1 impairs AvrRps4 and PopP2 recognition.

**Figure 1:**
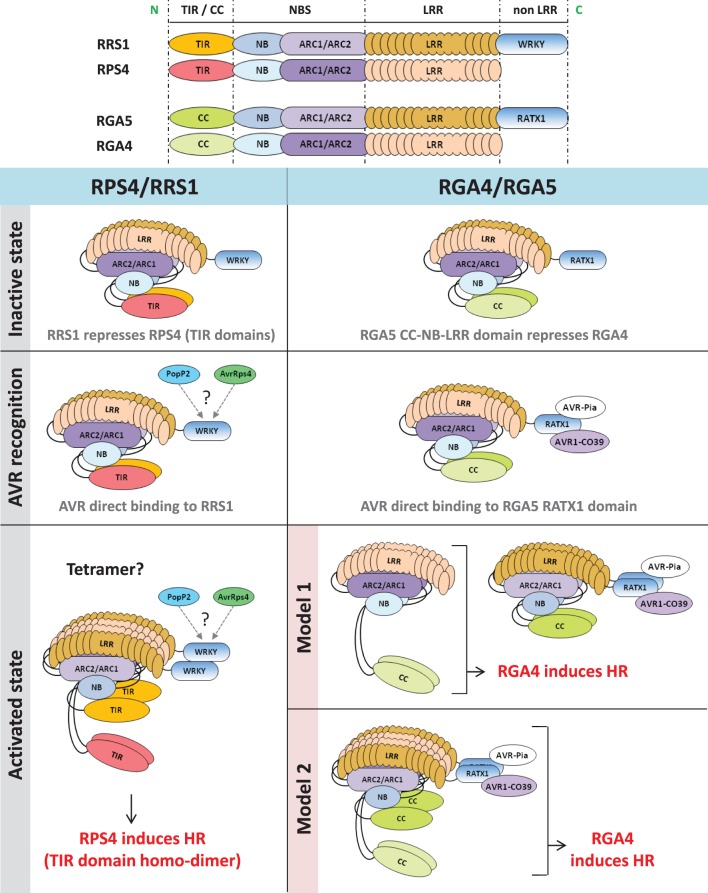
**Functional models of RPS4/RRS1 and RGA4/RGA5 NLR pairs**. In the inactive state, co-acting NLRs form a hetero-complex in which the signaling NLR (RPS4 or RGA4) is repressed by the AVR-receptor NLR (RRS1 or RGA5). After pathogen challenge, direct recognition of AVR proteins by the receptor NLR partner (RRS1 or RGA5) occurs. In the case of RGA5, AVR-binding involves the C-terminus containing the RATX1 domain while RRS1 interacts with PopP2 and AvrRps4 through its WRKY domain (Jones and Deslandes, personal communication). After AVR-recognition, NLR hetero-complexes are still present in both cases. Induction of HR by RPS4/RRS1 involves disruption of TIR domains hetero-interaction and subsequent homo-dimerization of RPS4 TIR domain. In the case of RGA4 and RGA5, we cannot rule out that induction of HR requires disruption of RGA4 and RGA5 hetero-complexes. Hence, two hypothetical models are proposed. According to the first model, HR is induced by RGA4 homo-complex freed from the RGA5/AVRs complex, whereas, in the second model, conformational changes within the RGA4/RGA5 hetero-complex would allow RGA4 to signal. N, N-terminal; C, C-terminal; TIR, Toll/interleukin1 receptor; CC, coiled coil; NBS, nucleotide binding site; NB, nucleotide binding; ARC, Apaf-1, R-protein and CED-4; LRR, leucine-rich repeats; HR, hypersensitive response.

Both AvrRps4 and PopP2 are recognized by direct binding to the RRS1 protein, which contains a C-terminal WRKY domain, but do not interact with RPS4 (Deslandes et al., 2003; Tasset et al., 2010; Williams et al., 2014). Interestingly mutation of the P-loop motif required for nucleotide binding abolishes the function of RPS4, consistent with the standard NLR activation model, while P-loop mutations do not affect RRS1 function, suggesting a specialized receptor function that does not require the formation of a nucleotide-dependent activation state (Williams et al., 2014). Thus, Williams et al. proposed a model in which RPS4 and RRS1 form an inactive hetero-dimer in the absence of pathogen recognition. Binding of the AVRs to RRS1 causes the disruption of the RPS4/RRS1 TIR domain hetero-dimer, allowing formation of a signaling competent RPS4 TIR domain homo-dimer (Figure 1). Since recognition of the AVRs does not seem to lead to disruption of the RRS1/RPS4 full-length hetero-complex, the activated state may be a tetramer. Thus, RRS1 and RPS4 function as a hetero-complex receptor with each partner having a different role: RPS4 acting as an inducer of disease resistance signaling and RRS1 acting as an effector binding receptor and repressor of RPS4 TIR signaling activity.

In the second case, Cesari et al. (2014) investigated the mode of action of RGA4 and RGA5, two rice CC-NLRs. Together, RGA4 and RGA5 are necessary and sufficient to mediate *Pia* and *Pi-CO39* resistances and recognize the *M. oryzae* effectors AVR-Pia and AVR1-CO39 (Cesari et al., 2013). Previous work showed that both effectors interact physically with RGA5, but do not bind to RGA4 suggesting that, similarly to RRS1, RGA5 acts as an AVR-receptor (Cesari et al., 2013). A domain at the C-terminus of RGA5 characterized by a heavy metal associated domain related to the cytoplasmic copper chaperone ATX1 from *Saccharomyces cerevisiae* (RATX1 domain) was identified as the AVR-binding domain in RGA5. Expression of the full length RGA4 protein, but not RGA5, in rice protoplasts and *Nicotiana benthamiana* leaves triggered cell death (Cesari et al., 2014). This autonomous activity of RGA4 was dependent on an intact P-loop motif and may be related to an unusual MHD motif within its NB domain in which the three usually highly conserved core amino acids are replaced by TYG. Mutational analysis showed that the glycine in this motif is crucial for the autoactivity of the protein as restoration of an aspartate residue at this position abolishes cell death. Yeast two-hybrid and co-immunoprecipitation assays indicate that RGA4 and RGA5 interact and form both homo- and hetero-complexes. These interactions are largely mediated by their CC domains although the RATX1 domain of RGA5 also self-interacts in yeast two-hybrid assay and could contribute to RGA5 homo-complex formation. Co-expression of RGA5 suppressed RGA4-mediated cell death indicating that formation of the RGA4/RGA5 hetero-complex might be crucial to regulate RGA4 activity in the absence of pathogen. Interestingly, the RATX1 domain of RGA5 is dispensable for RGA4 inhibition, showing that it probably serves primarily as an effector binding platform. Co-expression of AVR-Pia with RGA4 and RGA5 again triggered cell death. Hence, a functional model was proposed in which RGA4 acts as a signaling component regulated by its interaction with RGA5 which acts both as a repressor and a receptor that directly binds the AVR proteins (Figure 1).

From those discoveries, striking similarities are apparent between the RPS4/RRS1 and the RGA4/RGA5 functional models. Within a pair, distinct NLR proteins: (i) form homo- and hetero-complexes that are involved in important regulation processes both prior to and following pathogen recognition, (ii) cooperate to achieve one task, but are specialized and accomplish distinct functions within the complex (i.e., AVR-receptor or signal inducer), (iii) recognize multiple AVR proteins through direct binding to the “receptor” partner which possesses an unusual domain that is not conserved in other NLRs, (iv) rely on the N-terminal TIR or CC domain of the “signal inducer” NLR to translate recognition into activation of resistance responses. Recent analysis of the Pik-1 and Pik-2 CC-NLR pair showed that these proteins also form hetero-complexes through their CC domains (Zhai et al., 2014), with Pik-1 acting as a receptor mediating direct recognition of AVR-Pik whereas Pik-2 does not bind the effector but possesses HR signaling activity (Kanzaki et al., 2012; Cesari et al., 2013; Zhai et al., 2014). Thus, these functional models may be generally applicable to other NLR protein pairs.

## An integrated decoy model describes AVR recognition by NLR pairs

NLR proteins mediate recognition of AVR effectors either by direct physical interaction (Dodds and Rathjen, 2010) or in an indirect manner (Dangl and Jones, 2001; van der Hoorn and Kamoun, 2008). In cases of indirect recognition, the NLR responds to modifications of another plant protein induced by the AVR protein. This plant protein may be either the operational target of the effector, in which case it is called a “guardee” since it is guarded by the NLR (Dangl and Jones, 2001), or a mimic of the operational target in which case it is called a “decoy” (van der Hoorn and Kamoun, 2008).

In the case of NLR pairs, all studied examples show direct binding between AVR effectors and the NLR acting as an AVR receptor. AVR1-CO39 and AVR-Pia bind to RGA5, AvrRps4, and PopP2 interact with RRS1 and AVR-Pik binds to Pik-1 (Kanzaki et al., 2012; Cesari et al., 2013; Williams et al., 2014; Zhai et al., 2014). However, one striking feature of these NLR receptor proteins is that, apart from their conserved multidomain NLR architecture, they all contain additional unusual non-conserved domains (Table 1). Indeed, RRS1 possesses a C-terminal WRKY domain (Deslandes et al., 2002, p. 1) and RGA5 and Pik-1 both display a similar RATX1 domain (Okuyama et al., 2011; Cesari et al., 2013). In other known NLR pairs, one partner also contains an additional domain: a C-terminal protein kinase domain in Rpg5 (Brueggeman et al., 2008), an N-terminal TIR-NB-DUF640 in RPP2A (Sinapidou et al., 2004), a C-terminal NB domain in Prv (Brotman et al., 2012), and a second NB domain following the first one in RGA2 (Loutre et al., 2009). In Pi5-2 we identified a C-terminal domain containing a cleavage site (VPPFGEW, amino acids 1022 to 1028) for the *P. syringae* type III effector AvrRpt2, similar to that found in RIN4. This observation raises a central question: What could be the function of those specific non-conserved domains? Their striking diversity and the fact that they are not conserved in the majority of NLRs suggests that they do not play a critical role in signaling or regulation within the pair. This is consistent with the observation that RGA5, Pik-1 and RRS1 do not function directly in resistance signaling and that the RATX1 domain of RGA5 is not required to regulate RGA4.

**Table 1:** **Unusual domains in paired NLR R proteins**.

**NLR pair**	**Pathosystem (pathogen/host)**	**NLR with unusual domain**	**Unusual or additional domain**	**Location of the domain**	**References**
RRP2A RPP2B	*H. arabidopsidis*/Arabidopsis	RPP2A	TIR-NB-DUF640	N-terminus	Sinapidou et al., 2004
RRS1 RPS4	*R. solanacearum, P. syringae, C. higginsianum*/Arabidopsis	RRS1	WRKY	C-terminus	Gassmann et al., 1999; Deslandes et al., 2003; Birker et al., 2009; Narusaka et al., 2009
RGA4 RGA5	*M. oryzae*/rice	RGA5	RATX1	C-terminus	Okuyama et al., 2011; Cesari et al., 2013
Pik-1 Pik-2	*M. oryzae*/rice	Pik-1	RATX1	Between the CC and NB	Ashikawa et al., 2008; Yuan et al., 2011; Cesari et al., 2013; Zhai et al., 2014
Pi5-1 Pi5-2	*M. oryzae*/rice	Pi5-2	AvrRpt2-cleavage site	C-terminus	Lee et al., 2009
Fom-1 Prv	*F. oxysporum*, Papaya ring-spot virus/melon	Prv	NB	C-terminus	Brotman et al., 2012
RGA1 Rpg5	*P. graminis*/barley	Rpg5	Protein kinase	C-terminus	Wang et al., 2013
Lr10 RGA2	*P. triticina*/wheat	RGA2	NB	Between the CC and NB	Loutre et al., 2009

**Figure 2:**
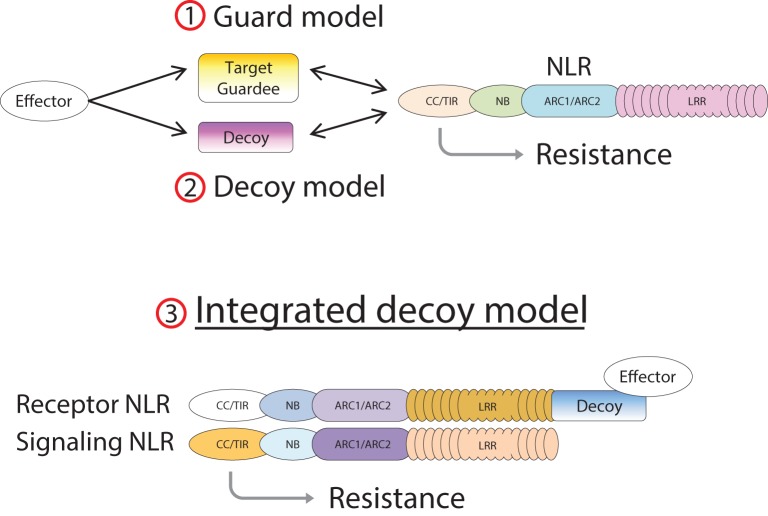
**Model of integrated decoys in NLR protein pairs**. Pathogen effectors target host proteins for manipulation in order to promote infection. Indirect recognition of these effectors occurs when these target proteins are guarded by host NLR proteins (1), or if duplicated target genes evolve into decoy proteins monitored by host NLRs (2). Alternatively the decoy may be integrated into the structure of the receptor component of an NLR pair (3), allowing AVR recognition by direct binding.

In the case of RGA4/RGA5, both AVR-Pia and AVR1-CO39 bind directly to the RATX1-containing domain at the C-terminus of RGA5 (Cesari et al., 2013). A similar RATX1 domain is also present in Pik-1 and this was shown to bind AVR-Pik (Kanzaki et al., 2012; Cesari et al., 2013; Zhai et al., 2014). However, in Pik-1, the RATX1 is located between the CC and the NB domains suggesting that this unusual AVR-binding domain has been acquired independently in the two CC-NLRs. In the light of these findings, we propose an “integrated decoy” model to explain effector recognition in these cases of paired NLRs (Figure 2). This is an extension of the decoy model (van der Hoorn and Kamoun, 2008) in which proteins that were initially targeted by pathogen effectors, have evolved into decoys guarded by NLRs. In this case however, the decoy is integrated in the R protein structure allowing direct recognition of AVR effectors. Support for the idea that the RATX1 domain present in RGA5 and Pik-1 could be a decoy comes from the observation that a similar domain is also present in the rice protein Pi21 which is required for full susceptibility to the rice blast fungus (Fukuoka et al., 2009). Thus, proteins containing this domain could be important virulence targets for *M. oryzae* effectors such as AVR1-CO39, AVR-Pia, and AVR-Pik. The C-terminal domains or motifs of RRS1 (WRKY), Pi5-2 (AvrRpt-cleavage site), and Rpg5 (protein kinase) all belong to families of proteins that have important roles in defense signaling (Pandey and Somssich, 2009; Deslandes and Rivas, 2012; Lin et al., 2013) supporting the hypothesis that they may represent integrated decoys. Consistent with this, recent unpublished work has suggested that AvrRps4 and PopP2 interact with the WRKY domain of RRS1 (Deslandes and Jones, personal communication). A key prediction of this model is that the virulence targets of these effectors are related to the recognition domains of the receptor NLR protein; RATX1 domain containing proteins in the case of AVR-Pia, AVR1-CO39 and AVR-Pik, and WRKY class transcription factors in the case of AvrRps4 and PopP2. Thus, the identification of the virulence targets of these effectors, which has not yet been reported, will be a critical test of the general validity of this model.

A genome search revealed that rice cultivar Nipponbare contains nine homologous gene pairs to RGA4/RGA5, which are all arranged as divergently transcribed genes with one exception arranged in a head-to-tail orientation. Interestingly, despite high sequence similarity in the CC-NLR domains, the RATX1 domain is not conserved among RGA5 rice homologs, nor in a wider set of RGA5 homologs identified in cereals, and in fact this C-terminal region is substituted in many cases by different protein domains that may also act as integrated decoys (Table 2, Supplemental Tables 1, 2). Consistent with this idea, many of these domains belong to protein families that are involved in disease resistance, such as protein kinases (i.e., MAPKs), WRKYs, or AvrRpt-cleavage site containing proteins. Likewise, Arabidopsis contains eight homologous pairs to RPS4/RRS1 (Narusaka et al., 2009), and a BLAST analysis performed with the RRS1 protein also identified many homologs in other plants in which the WRKY domain is replaced by various C-terminal domains, such as protein kinases, TIR, Zinc-finger, transcription elongation factor or different WRKY domains (Table 2, Supplemental Tables 3, 4). Blast2GO analysis of the C-terminal domains of the RGA5 and RRS1 homologs showed very similar Gene Ontology (GO) enrichment results. In terms of biological processes, both sets show a significant enrichment for “defense response” (Supplemental Figure 1) while in terms of molecular function, they both show a significant enrichment for catalytic activities such as “kinase activity” or “phosphatase activity” and for binding activity such as “DNA binding” (Supplemental Figure 2). This observation suggests that the protein domains that have been integrated in the structure of RGA5 and RRS1 homologs share similar functions and are involved in similar biological processes, particularly defense responses.

**Table 2:**
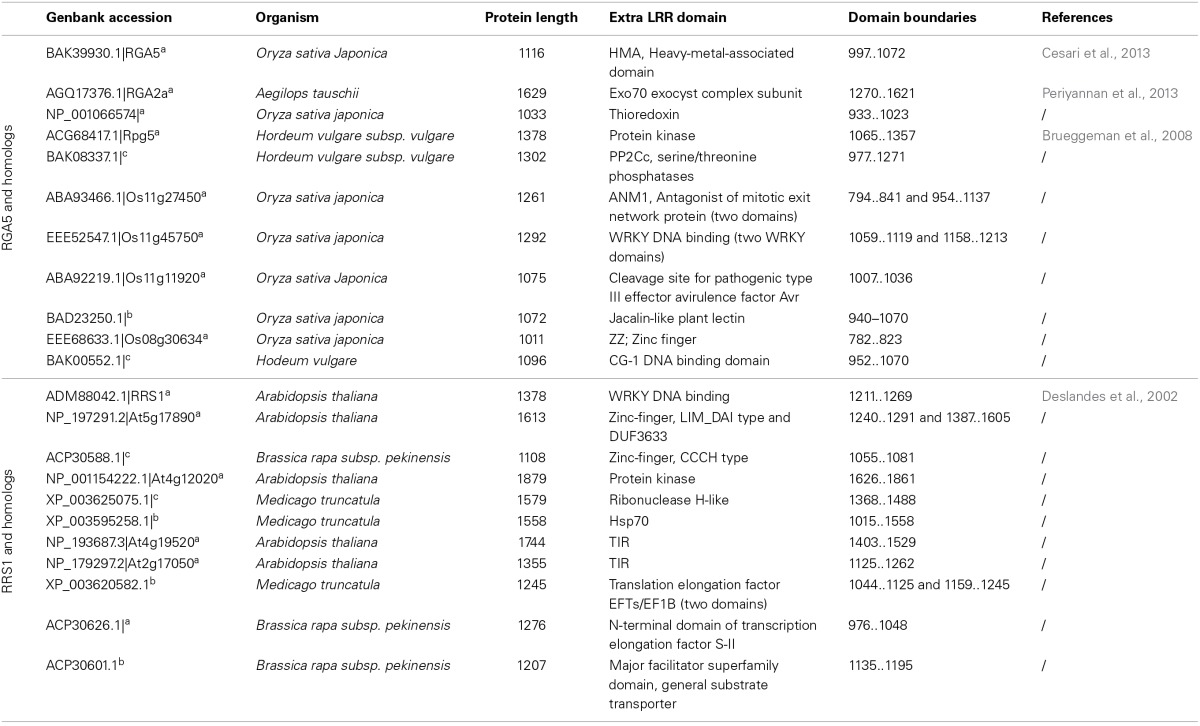
**Diversity of integrated decoys in selected RGA5 and RRS1 homologs**.

*^a^NLR proteins coded by genes that occur together with an adjacent NLR homolog to RGA4 or RPS4*.

*^b^NLR proteins coded by genes that occur singly*.

*^c^Genome context unknown*.

These observations suggest that diverse protein domains have been integrated in the NLR protein acting as a receptor in order to sense and physically bind AVR-effectors. In evolutionary terms, duplication of paired NLR genes may be followed by acquisition of new domains through random genome re-arrangements, with selection favoring chimeric NLR-decoy proteins that confer novel resistance phenotypes. Why these events seem to occur predominantly in paired NLRs, rather than in single NLRs is an intriguing question. Perhaps this architecture offers a unique functional model to exploit this integration because the mode of action only requires that interaction of the AVR with the receptor NLR disrupts suppression of the signaling NLR.

Under the integrated decoy model the “direct” vs. “indirect” recognition dichotomy is no longer appropriate to describe AVR recognition by NLR pairs, which have apparently evolved a direct recognition function via incorporation of a decoy molecule into their structure. Nevertheless, other examples of direct recognition, such as those mediated by the R proteins RPP1 in Arabidopsis, L6 and M in flax or N in tobacco, involve single NLR proteins with no additional domains (Dodds et al., 2006; Ueda et al., 2006; Catanzariti et al., 2010; Krasileva et al., 2010). As observed for these directly interacting single NLRs, the decoy component of the NLR pair may be in an evolutionary arms race with the corresponding effectors. Consistent with this, analysis of polymorphisms within Pik-1 indicates that diversifying selection occurs at the RATX1 domain and that these polymorphisms are responsible for recognition specificity of AVR-Pik alleles by the different Pik-1 alleles (Kanzaki et al., 2012).

## Striking similarities and differences between plant and animal co-acting NLRs

In an interesting parallel, several cytosolic mammalian NLR immune receptors have also been shown to function in heteromeric complexes (von Moltke et al., 2013). One of them, NAIP5 (NLR family, apoptosis inhibitory proteins 5), was shown to be essential to restrict the pathogenic bacteria *Legionella pneumophila* in mice macrophages (Beckers et al., 1995; Dietrich et al., 1995) and to respond to flagellin, inducing a caspase-1 dependent pyroptotic cell death (Lightfield et al., 2008; Bergsbaken et al., 2009). However, NAIP5 lacks a signaling CARD (caspase activation and recruitment domain) and caspase-1 activation relies on a second member of the NLR family, called NLRC4, which contains an N-terminal CARD (Amer et al., 2006; Franchi et al., 2006; Miao et al., 2006).

Two elegant studies shed light on the role and function of NAIP5 and NLRC4 upon flagellin perception (Kofoed and Vance, 2011; Zhao et al., 2011). Co-expression of flagellin with NLRC4 and NAIP5 in human cells lacking NAIP5 and NLRC4 homologs results in the formation of an inflammasome, a high molecular complex that contains the three proteins and activates the caspase-1 signaling pathway. Within the complex, flagellin interacts directly and specifically with NAIP5, but not with NLRC4. In this system, NAIP5 acts as the direct receptor for flagellin while NLRC4 orchestrates downstream signaling responses.

Interestingly, the NLCR4 inflammasome also responds to another conserved bacterial motif present in the type III secretion system of many bacterial pathogens: the inner rod protein (i.e., PrgJ in *Salmonella* and BsaK in *Burkholderia*) (Miao et al., 2010). However, NAIP5 is not required for PrgJ-mediated NLRC4 activation (Lightfield et al., 2011) and screening other members of the NAIP family revealed that NAIP2 binds directly and specifically to BsaK and PrgJ (Kofoed and Vance, 2011; Zhao et al., 2011). Co-expression of NAIP2, PrgJ and NLRC4, results in the formation of an inflammasome and caspase-1 mediated signaling activation.

Thus, similarly to plant co-acting NLRs, NAIPs, and NLRC4 act as a dual immune receptor in which one partner (NAIP) specifically recognizes and binds to pathogen molecules whereas the other partner (NLRC4) translates immune signaling. However, *Naip* and *Nlrc4* genes are not co-located in the mouse genome, as seen for the plant NLR gene pairs. In addition, NAIP proteins are involved in the recognition of PAMPs whereas plant NLR proteins recognize specific effectors. Another striking difference is that NAIPs are monomeric in the absence of ligand and associate with NLRC4 only upon ligand recognition (Halff et al., 2012; Tenthorey et al., 2014) whereas the plant paired NLRs associate before and after effector recognition (Cesari et al., 2014; Williams et al., 2014). Thus, NLRC4 seems to act as a downstream signaling partner for various NAIP receptors. In contrast, no other NLRs have been shown to bind to RGA4 or RPS4, so they do not appear to be common signaling partners, but rather act as part of a specific receptor complex. On the other hand two plant NLRs, Arabidopsis ADR1 and tomato NRC1 have been described to act as a signaling hub or NB-LRR helper for the function and defense signaling activation of certain immune receptors (Gabriëls et al., 2007; Roberts et al., 2013), and thus could function in a similar manner to NLRC4. However, there is not yet any evidence that ADR1 or NRC1 interact physically with other NLRs. It is possible that the genetically paired NLRs may have evolved from such helper interactions through physical coupling of co-evolved receptor and helper NLR genes.

## Concluding remarks and future directions

The molecular characterization of the first *NLR* genes was achieved 20 years ago with the cloning of *RPS2* and *N* (Bent et al., 1994; Mindrinos et al., 1994; Whitham et al., 1994). Since then, remarkable progress in our understanding of NLR proteins' mode of action have been made, unraveling a high level of complexity in the way these receptors operate to perceive pathogen effectors in plant cells. Both “direct” and “indirect” AVR-recognition have been observed and the “integrated decoy” model described here could be regarded as an intermediate between these recognition modes. This model implies that some instances of direct recognition are derived from guard/decoy type indirect recognition. Numerous potential integrated decoys occur in RRS1 and RGA5 homologs and if they represent domains targeted by effector proteins, their identification and analysis should provide valuable information about cellular processes targeted by pathogens during infection. In addition, integrated decoys could also be used as baits to screen for interacting AVR proteins. Ideally, the widespread nature of integrated decoys in NLR protein pairs should pave the way toward R protein engineering allowing effector targets to be fused to a receptor NLR operating within a pair to create an “effector trap.”

### Conflict of interest statement

The authors declare that the research was conducted in the absence of any commercial or financial relationships that could be construed as a potential conflict of interest.

## Supplementary material

Supplemental Figure 1 **Domains integrated in RGA5 and RRS1 homologs are involved in similar biological processes, particularly defense responses. (A)** Five hundred homologs of RGA5 were identified by Blastp against the NCBI nr database. Their sequences were aligned and a cut-off between the conserved N-terminus and the variable C-terminus was defined in the multiple alignment. C-terminal sequences (after the cut-off) were analyzed by Blast2GO and total number of GO annotations corresponding to each biological process is displayed. **(B)** Same procedure as in **(A)** was applied for RRS1 homologs (cut-off 1). GO, gene ontology; BP, biological process; #seqs, number of sequences; #GO, GO terms.

Supplemental Figure 2 **Domains integrated in RGA5 and RRS1 homologs show a significant enrichment for similar molecular functions. (A)** Blast2GO analysis for RGA5 homologs' C-terminal domains (molecular function). **(B)** Same as in **(A)** for RRS1 homologs' C-terminal domains (cut-off 1). GO, gene ontology.

Supplemental Table 1 **Manually identified C-terminal domains in RGA5 homologs**.

Supplemental Table 2 **Interproscan identified C-terminal domains in RGA5 homologs**.

Supplemental Table 3 **Interproscan identified C-terminal domains in RRS1 homologs (cut-off L850)**.

Supplemental Table 4 **Interproscan identified C-terminal domains in RRS1 homologs (cut-off Y894)**.
