# Supplementary material for: A novel conserved mechanism for plant NLR protein pairs: the “integrated decoy” hypothesis
Source: Front Plant Sci. 2014 Nov 25;5:606. doi: 10.3389/fpls.2014.00606 (PMC4246468; doi:10.3389/fpls.2014.00606)
Supplement: Supplemental Figure 1 — Domains integrated in RGA5 and RRS1 homologs are involved in similar biological processes, particularly defense responses. (A) Five hundred homologs of RGA5 were identified by Blastp against the NCBI nr database. Their sequences were aligned and a cut-off between the conserved N-terminus and the variable C-terminus was defined in the multiple alignment. C-terminal sequences (after the cut-off) were analyzed by Blast2GO and total number of GO annotations corresponding to each biological process is displayed. (B) Same procedure as in (A) was applied for RRS1 homologs (cut-off 1). GO, gene ontology; BP, biological process; #seqs, number of sequences; #GO, GO terms. [file Presentation1.PPTX]

## Slide 1
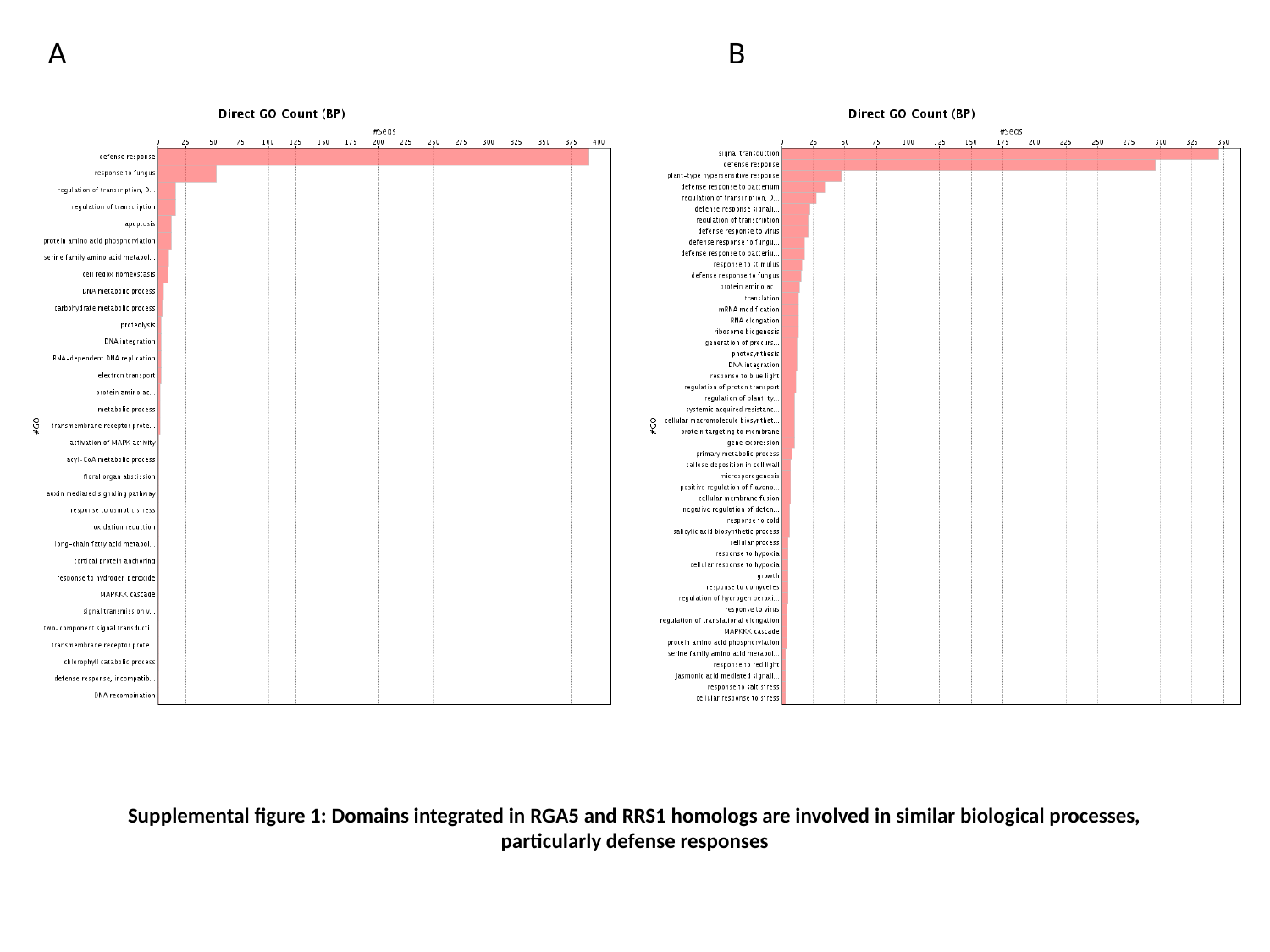

A
B
Supplemental figure 1: Domains integrated in RGA5 and RRS1 homologs are involved in similar biological processes, particularly defense responses
