# Supplementary material for: A novel conserved mechanism for plant NLR protein pairs: the “integrated decoy” hypothesis
Source: Front Plant Sci. 2014 Nov 25;5:606. doi: 10.3389/fpls.2014.00606 (PMC4246468; doi:10.3389/fpls.2014.00606)
Supplement: Supplemental Figure 2 — Domains integrated in RGA5 and RRS1 homologs show a significant enrichment for similar molecular functions. (A) Blast2GO analysis for RGA5 homologs' C-terminal domains (molecular function). (B) Same as in (A) for RRS1 homologs' C-terminal domains (cut-off 1). GO, gene ontology. [file Presentation2.PPTX]

## Slide 1
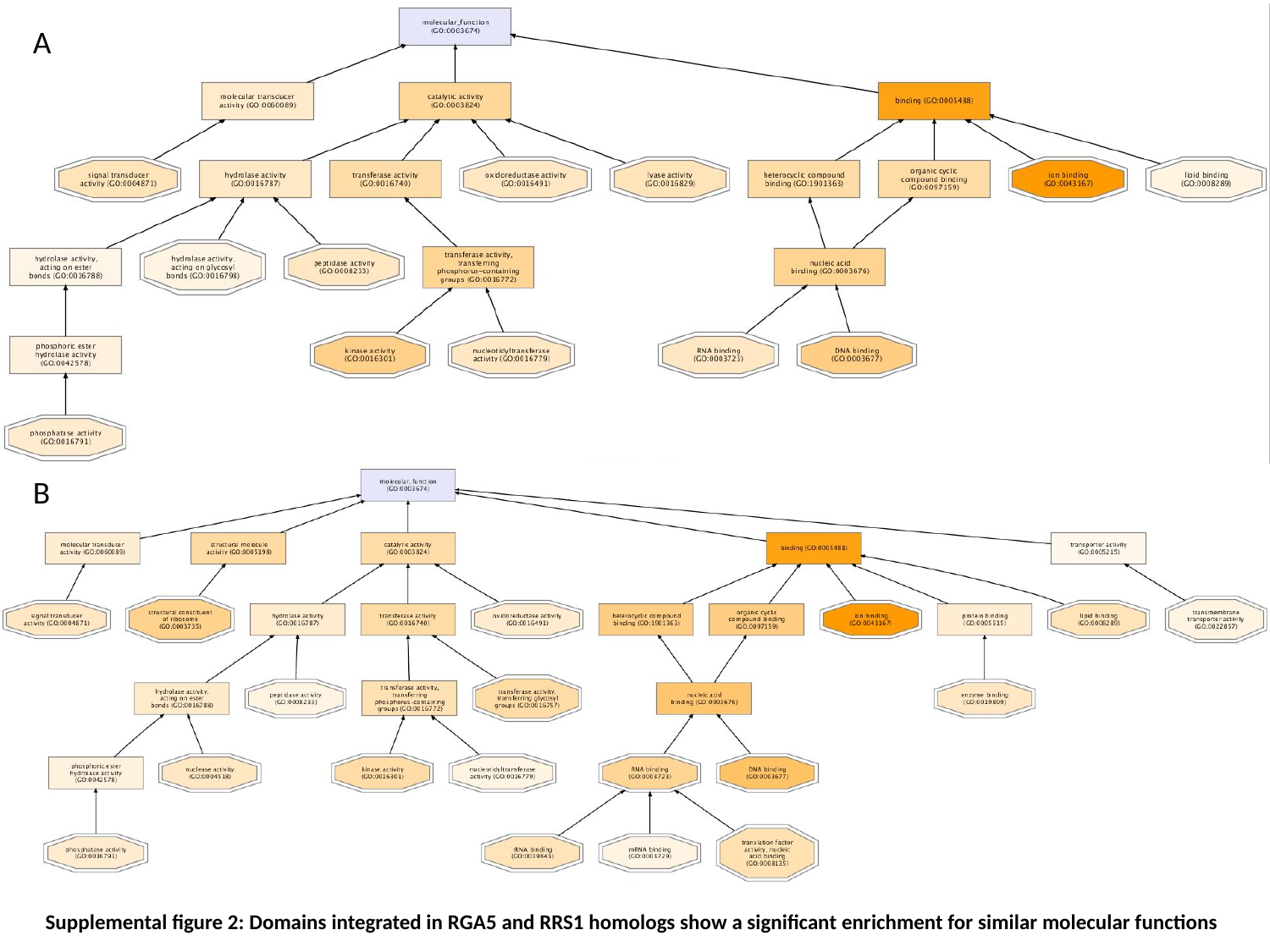

A
B
Supplemental figure 2: Domains integrated in RGA5 and RRS1 homologs show a significant enrichment for similar molecular functions
